# Combining computer‐based rehabilitative approach with tDCS for recovering of aphasia: Implications from a single case study

**DOI:** 10.1002/ccr3.8928

**Published:** 2024-05-22

**Authors:** Marianna Contrada, Federica Scarfone, Antonella Iozzi, Simone Carozzo, Martina Vatrano, Maria Grazia Nicoletta, Giuseppe Nudo, Maria Quintieri, Paolo Tonin, Antonio Cerasa

**Affiliations:** ^1^ S. Anna Institute Crotone Italy; ^2^ Institute for Biomedical Research and Innovation (IRIB) National Research Council of Italy (CNR) Messina Italy; ^3^ Department of Pharmacy, Pharmacotechnology Documentation and Transfer Unit, Preclinical and Translational Pharmacology, Health Science and Nutrition University of Calabria Rende Italy

**Keywords:** aphasia, language comprehension, stroke, tDCS, VRRS cognitive treatment

## Abstract

We present a case of a single left hemisphere temporal–parietal stroke with subacute global aphasia and severe verbal apraxia and moderate dysphagia. The patient underwent a combined transcranial direct current stimulation (tDCS) over the left dorsolateral prefrontal cortex (DLPFC) and language stimulation with Virtual Reality Rehabilitation System (VRRS). Patient was treated in a 1‐h session, for 5 days a week, for 4 consecutive weeks. After treatment, evident improvements in the comprehension of oral and written language, swallowing abilities, and caregiver burden were detected. Power spectrum analysis of EEG data revealed significant enhancements of *θ*, *α*, and *β* waves from baseline to follow‐up. These preliminary results seem to confirm the reliability of the tDCS translational application in conjunction with computer‐based cognitive treatment for language disorders in a patient with stroke‐induced aphasia.

## 1 INTRODUCTION

Intense language training lasting many hours per week is now considered and recommended as a critical predictor for successful language outcomes in stroke patients.^1^ Unfortunately, most aphasic individuals are unable to receive the recommended amount of training estimated due to economic constraints in the health systems or barriers to access services.^2^ Therefore, in order to enhance the language recovery process, new rehabilitation techniques that serve as a complement to or a replacement for conventional procedures are urgently required.

In the past 10 years, a growing body of research has supported the use of noninvasive brain stimulation (NIBS) techniques to improve aphasia patients' long‐term recovery.^3^ In aphasia rehabilitation, transcranial direct‐current stimulation (tDCS) is one of the most popular NIBS treatments. It has been demonstrated to be useful in encouraging a full recovery from a brain injury, both immediately and over time.^4^ The primary advantage of tDCS is in its potential for minimal adverse effects when used appropriately and carefully. Treatments like speech and language therapy have been shown to work better when combined with tDCS to improve the recovery of the affected functions.^4^, ^5^ Despite a large employment of the combined tDCS and cognitive approach to recover language disorders in aphasic patients,^3^ the real gains varied from modest to moderate, and recovery is frequently restricted to the treated items.^4^ In the previous literature, many studies coupled tDCS application with logopedic traditional treatment.^6^ In this study, we sought to demonstrate the effectiveness of a new combination of non‐invasive neurostimulation over the left dorsolateral prefrontal cortex (DLPFC) with a well‐known and validated computer‐based cognitive protocol: Virtual Reality Rehabilitation System (VRRS).^7^ Since that tDCS over the DLPFC alone has been demonstrated to be effective in improving language abilities,^8^ as well as VRRS treatment,^9^ we hypothesized that the combination of these methods can provide a boosting effect on a patient with severe aphasia.

## 2 CASE HISTORY

**FIGURE 1:**
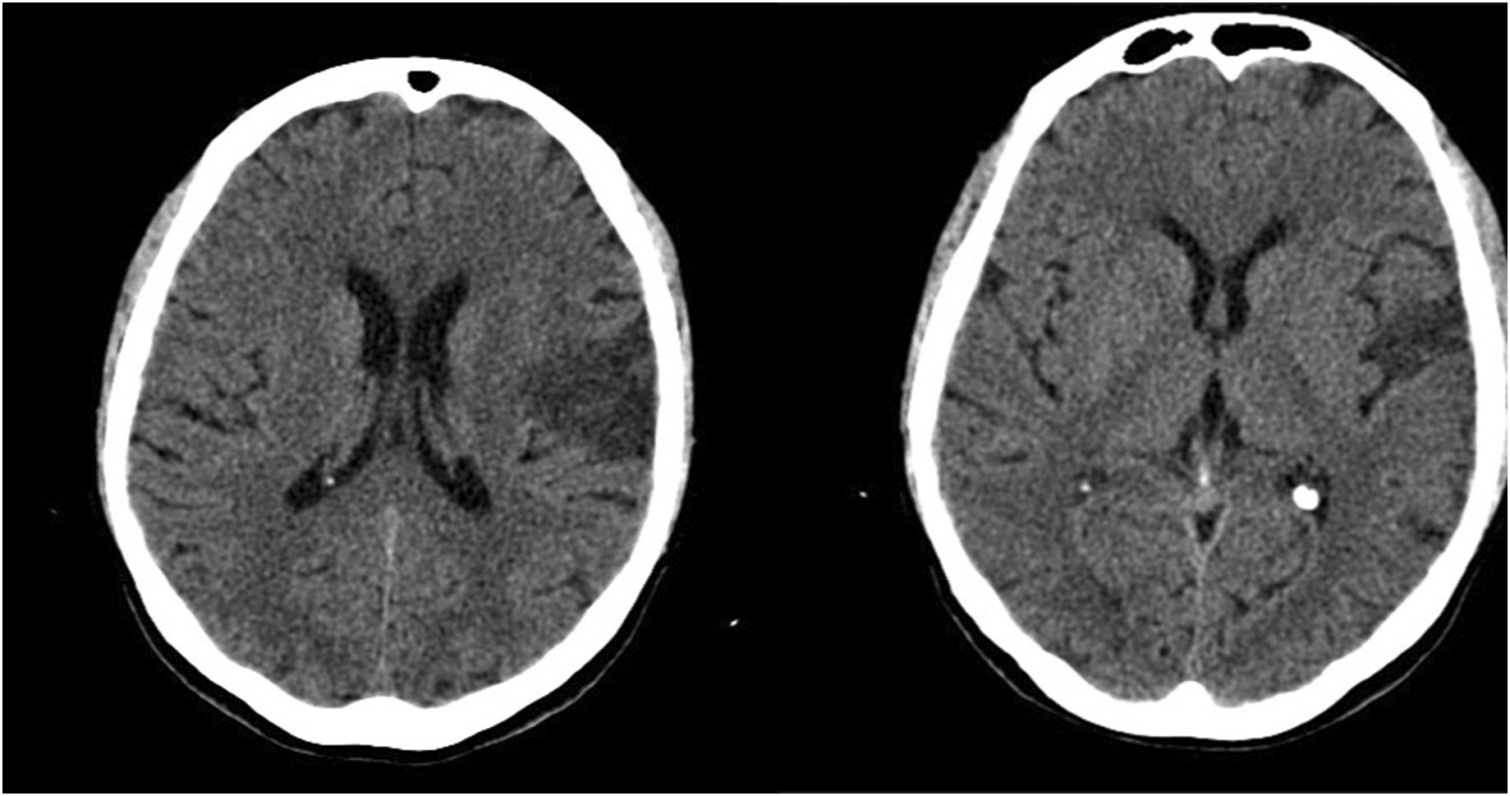
Computerized tomography showing left ischemic cerebral infarction.

We present a 62‐year‐old right‐handed female stroke case (5 years of education). The patient was subsequently admitted on February 11, 2021, to the intensive rehabilitation unit (IRU) at the S. Anna Institute in Crotone, Italy, and released on May 19, 2021. Three months before enrolling in the research, she suffered a single left hemisphere stroke (ischemic cerebral infarction in the lateral sulcus) (Figure 1). At admission in IRU, the patient was characterized by a subacute global aphasia with severe verbal apraxia and moderate dysphagia. The participant gave written informed consent. The study was approved by the Central Area Regione Calabria of Catanzaro (Protocol n. 346. October 21, 2021).

## 3 METHODS

### 3.1 Procedure

**FIGURE 2:**
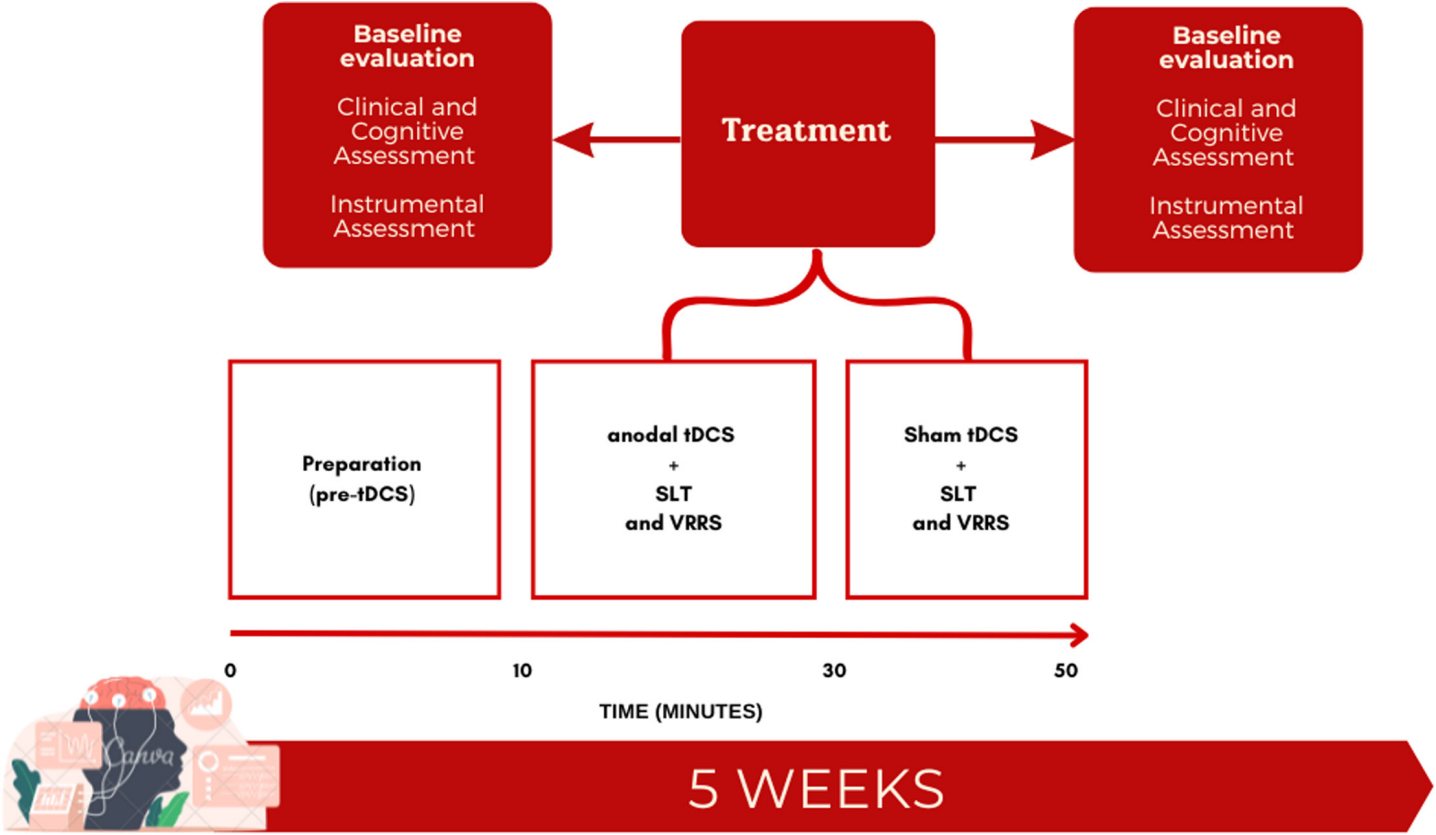
Experimental procedure. The patient underwent clinical, cognitive, and instrumental evaluation before and after 4 weeks of combined tDCS+cognitive treatment. The tDCS stimulation lasted 50 minutes including preparation phase, anodal tDCS stimulation, followed by sham tDCS. During tDCS stimulation, the patient underwent cognitive intervention. SLT, standard language therapy; VRRS, Virtual Reality Rehabilitation.

During the neurorehabilitation period, the patient underwent a combined tDCS and cognitive treatment lasting five times a week for 1‐h sessions for 4 consecutive weeks. The tDCS procedure was performed according to Marangolo et al.^10^ Before (T_0_) e and after treatment (T_1_), the patient underwent clinical, cognitive and instrumental evaluations (Figure 2). The protocol included the use of electroencephalographic examination (EEG) to examine brain waves activity before and after treatment, to evaluate if intensive rehabilitation would facilitate inter‐ and intra‐hemispheric reorganization.

Two blocks of tDCS stimulation (anodal and sham) were performed together with cognitive treatment. The tDCS was activated during the first 20 min of the cognitive treatment (anodal tDCS) after the electrodes were mounted (preparation phase). Following this, the mounting was maintained while the clinicians turned off the tDCS (sham tDCS) and the treatment proceeded (Figure 2). The participant was unaware of tDCS phases. Behavioral intervention includes both conventional speech and language therapy (SLT) together with advanced computer‐based speech recovery tasks made by Virtual Reality Rehabilitation System (VRRS) tool (Khymeia Group. Noventa Padovana, Italy; https://khymeia.com/it/).

### 3.2 tDCS intervention

The tDCS stimulation was carried out by means of BrainSTIM Direct Current Stimulator device (http://www.brainstim.it/). Two 5 × 5 cm = 25 cm^2^ electrodes were inserted into two sponge pockets, both soaked in saline to adjust the impedance. After, they were applied to the patient's scalp. The “active electrode,” referred to as the “anode,” was placed on the site above the cortical target, the left DLPFC (F3 site of the International 10–20 EEG system) while the “reference electrode,” referred to as the “cathode,” was placed on the contralateral zone, the supraorbital zone (Fp2 site of the International 10–20 EEG system), to deliver an anodic current on the left DLPFC. For the first 20 min of the treatment, the stimulation level was set to 2 mA. After that, it was turned off for the final 20 min. The combined cognitive and tDCS treatment was carried out for 20 sessions (5 consecutive days per week for 4 consecutive weeks) always at the same time of day.^10^, ^11^ Safety guidelines were followed accurately, and the participant was asked to report any discomfort or discomfort sometimes felt during the sessions.

### 3.3 Cognitive intervention

The cognitive intervention was performed with VRRS tool in combination with traditional speech language intervention.^12^ In particular:

1. The advanced VRRS intervention was focused on tasks eliciting comprehension, pragmatic‐articulation, phonological and semantic/lexical abilities. The planned logopedic approach was delivered by conventional vis‐à‐vis approach (exercises for the semantic‐lexical components, pragmatic component, and comprehension) and by using the VRRS digital speech therapy module, which includes an extensive set of exercise data organized by activity domain for written naming and respiratory education exercises. The module includes the possibility of using the K‐Space sensor to enable patients with motor difficulties to perform this kind of exercises: General Actions; Written naming; Phonation and Semantic recognition.
2. The traditional speech intervention consisted of exercises in five specific domains: Deficits of Comprehension; Praxic‐articulatory deficits; Treatment of phonological deficit; Treatment of semantic‐lexical deficits; and Treatment of morphosyntactic deficits.

### 3.4 Clinical and neuropsychological assessment

A complete motor, clinical, neuropsychological, and psychological evaluation was conducted before and after treatment. In particular, the post‐treatment testing was conducted the day after the treatment was finished on the two next days. For the assessment of the motor functions, we used the well‐known standard clinical scales: Barthel Index (BI) for the evaluation of daily activities, the Motricity Index scale for assessing motor functions, the Trunk Control Test (TCT) to assess trunk control and recovery of walking ability, and the Visual Analogic Scale (VAS) for pain perception assessment.

For the assessment of language, the Italian version of Aachen Aphasia Test (AAT) to assess phonological, lexical, semantic, and syntactic aspects of language; and the Communication Scale Boston Diagnostic Aphasia Examination, (BDAE) to assess spontaneous speech, listening comprehension, oral production, written language comprehension, and writing were used. To determine the dysphagia severity and the risk of aspiration in acute stroke patients, the Gugging Swallowing Screen (GUSS) was used. Apraxia was assessed using the Test for Bucco linguofacial Apraxia and by Ideomotor Apraxia Test.

Finally, a psychological and caregiver burden assessment was carried out using the Caregiver Burden Inventory scale (CBI). Neuropsychological assessments were blindly performed by two experienced clinicians (M.C. and F.S.).

### 3.5 Instrumental assessment (EEG)

The EEG was composed with a baseline lasting 15 min in two time windows: T_0_ and T_1_. The EEG recording was made at 256 Hz by EBNeuro device in the electrophysiology laboratory in the absence of noise. The subject was sitting comfortably in an armchair, in a dark room without possible acoustic or visual sensory stimuli. The EEG recordings were carried out at the beginning and after the treatment before neuropsychological evaluations. The acquisitions were made with a 19 channels cap with sintered Ag‐AgCl ring electrodes. Recording impedances were kept <5 kΩ. The signal was recorded with common reference and filtered between 1 and 45 Hz. EEG signals were extracted and analyzed by EEGLAB and visually controlled for artifacts. After artifacts remotion by independent component analysis (ICA) method, the power spectrum density (PSD) of theta (4–7 Hz), alpha (8–12 Hz), and beta (13–30 Hz) were extracted and expressed in dB (10log10 (μV^2^/Hz)).

### 3.6 Statistical analysis

Statistical analyses were performed by SPSS statistical software v.17.0 (SPSS Inc., Chicago, IL, USA) and R Language v.4.0.3 (R Foundation for Statistical Computing, Vienna, Austria). The Wilcoxon exact test was used to compare baseline and follow‐up EEG *θ*, *α*, and *β* PSD dataset. A significant threshold of *p* < 0.05 was established. For clinical and neuropsychological data, no statistical analysis was performed, but relevance of data is discussed referring to cut‐off criteria.

## 4 CONCLUSION AND RESULTS

The participant did not report any side effects or contraindications during tDCS treatment. Overall, the patient showed a relevant improvement in the right‐side motor abilities (Table 1). From a functional and motor perspective, the patient's full dependence on assistance with activity daily living was indicated by the first score on the BI. Following therapy, the patient virtually recovered her functional independence. The Motricity Index Scale score indicates that the patient regained good lower extremity functioning in the damaged limb (the right), whereas a trend toward full motricity recovery was noted in the upper extremity. The results of the TCT indicated a relevant improvement, whereas the VAS assessment indicated an absent improvement in the perception of pain severity.

**TABLE 1:** Clinical assessment after combined tDCS+cognitive treatment for aphasia.

Motor functions				
	Baseline		Follow‐up	
	Score	Deficit	Score	Deficit
Barthel Mobility Index	15/100	Severe	80/100	Mild
Motricity Index Scale				
Right upper extremity	0/100	Severe	28/100	Moderate
Right lower extremity	0/100	Severe	75/100	Mild
Left upper extremity	99/100	Norm	99/100	Norm
Left lower extremity	99/100	Norm	99/100	Norm
Trunk control test scale	25/100	Severe	100/100	Norm
Visual analogic scale	3/10	Mild	1/10	Mild
Communication				
BDAE	1	Severe	3	Severe
Dysphagia				
GUSS	11	Moderate	19	Mild
Praxia				
Buccolingual facial praxia test	6	Moderate	10	Moderate

Abbreviations: BDAE, Boston Diagnostic Aphasia Examination; GUSS, Gugging Swallowing Screen.

Assessing motor functions specifically related to language abilities, we detected an improvement in swallowing abilities (Table 1). At the neuropsychological level, relevant enhancement in the comprehension of oral (i.e., Token Test) and written language (Table 2), as well as in caregiver burden (Table 3) were also detected. However, the severity of the disease prevented the administration of the subitems about the written language, naming, and token test (Table 2) before therapy. Tests for written language and the token test could be given after therapy, and while the results clearly demonstrated a severe deficiency, they also qualitatively suggest progress, as evidenced by the improvement from the N/A level before treatment to the score of 1 for written language and 7 for the token test, after treatment, whereas no significant improvement was detected after treatment for naming. Finally, the Time Burden (the caregiver's time with the family member is reduced), Evolutionary Burden (the caregiver's perception of being excluded from opportunities and peers), and Physical Burden (feelings of fatigue and health problems of a somatic nature) scores decreased from pre‐ to post‐treatment, indicating a decrease in caregiver burden.

**TABLE 2:** Neuropsychological assessment before and after combined tDCS+cognitive treatment for aphasia.

Test	Total range by subtest	Score	Baseline			Score	Follow‐up		Deficit
AAT			T‐score	SN	Deficit		T‐score	SN	
Spontaneous speech	0–5	0	0	0	*Severe*	0	0	*0*	*Severe*
Token test	0–50	N/A	N/A	N/A		6	63	*7*	*Mild*
Repetition	0–150	11	33	2	*Severe*	8	31	*1*	*Severe*
Written language	0–90	N/A	N/A	N/A		0	31	*1*	*Severe*
Naming	0–120	N/A	N/A	N/A		N/A	N/A	N/A	
Oral comprehension	0–60	37	42	3	*Severe*	48	54	*6*	*Mild*
Written comprehension	0–60	17	38	3	*Severe*	21	50	*4*	*Moderate*

*Note*: Raw values were converted in T and SN scores following the AAT Italian normative scores.

Abbreviations: AAT, Aachen Aphasia Test; N/A, Not applicable for severity of disorder; SN, standard Nine.

**TABLE 3:** Psychological assessment before and after combined tDCS+cognitive treatment for aphasia.

Caregiver burden test	Baseline	Follow‐up
Total score	24	19
Subitem Time Burden—T	7	6
Subitem Evolutionary Burden—S	5	2
Subitem Physical Burden—F	6	5
Subitem Social Burden—D	4	4
Subitem Emotional Burden—E	2	2

**FIGURE 3:**
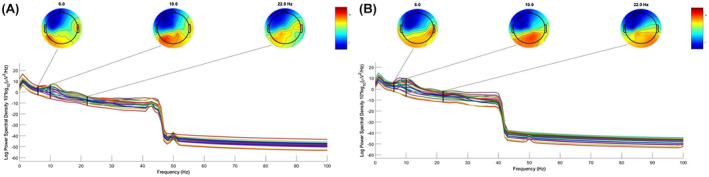
Power spectrum density and topographic plots from 1 to 45 Hz before (A) and after (B) treatment.

Moreover, power spectrum analysis of EEG data revealed significant enhancements of θ, α, and β waves from baseline to follow‐up (Figure 3, Table 4). Overall, the level of electrical alterations in the EEG remains “Medium” in both phases (Figure 3A,B), with a basic rhythm that increases slightly in frequency (from 10–11 c/s to 11–12 cs) and in amplitude (from 15–20 to 20 microvolts) and the constant presence of slow waves predominantly affecting the left hemisphere.

**TABLE 4:** EEG‐related mean frequency bands.

Cerebral activity				
EEG waves	Baseline	Follow‐up	Wilcoxon‐value	*p*‐Value
Theta 4–7 Hz	2.8	4.03	−3.1	<0.001
Alpha 8–12 Hz	1.62	3.12	−3.02	<0.001
Beta 13–30 Hz	−5.65	−3.81	−3.14	<0.001

## 5 DISCUSSION

We showed that a patient with subacute global aphasia achieved meaningful clinical (swallowing), cognitive (oral and written comprehension), and psychological (caregiver burden) gains by combining computer‐based language treatment with anodal tDCS over the left DLPFC. Moreover, the presence of significant changes in EEG θ, α, and β activities after the treatment suggests the presence of neural plasticity changes.

tDCS has typically facilitated a better recovery of the damaged language functions when used in conjunction with conventional behavioral therapies compared to offline tDCS.^13^ However, contrasting protocols of stimulation by tDCS emerge among the various scientific evidence including different brain areas of stimulation, such as the inferior frontal gyrus^14^ or superior temporal cortex.^15^ It has recently been demonstrated that anodal tDCS over the DLPFC may also increase M1 excitability.^16^ The DLPFC and M1 are functionally linked through many circuits, supporting the idea that DLPFC stimulation could help with motor rehabilitation or motor components of language disorders. This could explain because we also found improvement in dysphagic symptoms.

The use of a well‐known virtual system for cognitive rehabilitation is the second novel aspect of our research. The impact of VRRS for language recovery is in line with recent claims seeing language faculty as represented in a multimodal dimension where word semantics contain sensorimotor properties, which rely on primary motor and somatosensory networks.^8^ Using a VRRS to recover language functions remotely at home, Maresca et al.^7^ confirmed the key role of this kind of advanced method for patients with aphasia. In particular, after 6 months of exercises similar to those employed in our study, these authors reported significant improvements in 30 patients with aphasia of several language functions (including naming, comprehension, repetition, reading).

Finally, we also detected a relevant increase in the mean frequency of EEG θ, α, and β activities after treatment. This finding agrees with previous studies demonstrating that anodal tDCS of the left DLPFC induced relevant EEG changes in *θ* as well as *β* waves per se.^17^ In patients with aphasia, an increase of *θ* bands in the frontal left hemisphere has also been described after tDCS of the prefrontal cortex.^18^

Some limitations need to be acknowledged. Firstly, the absence of a defined tool for evaluating the negative effects of tDCS, which in this case is only supported by nonverbal self‐reports. Considering the subacute nature of this clinical case, it remains to be determined the amount of spontaneous recovery and if a cathodal tDCS contralateral frontal stimulation, given the size of the lesion, can be also considered. Lastly, the absence of a tDCS methodological assessment is the most significant constraint. We are unable to determine whether the documented clinical benefit is attributable to the use of sham stimulation in conjunction with a‐tDCS therapy or to the active treatment alone. However, it should be considered that anodal tDCS as well as sham tDCS of the left DLPFC induced significant and similar increases in the theta and alpha bands.^15^

To summarize, we show that an individual with subacute post‐stroke global aphasia can benefit greatly from the use of anodal tDCS over the DLPFC area in conjunction with advanced computer‐based speech and language treatment to improve their written and spoken comprehension language abilities. Notwithstanding the evident constraints of a case report study, our study highlights the importance of technology‐assisted initiatives to encourage new advanced treatments for aphasic patients and the practical implications of these findings are directly relevant to routine clinical practice. Some points remained to be determined: (a) whether the reported clinical recovery continues for a longer amount of time; (b) whether using sham stimulation in addition to a‐tDCS therapy accounts for it or if the active treatment alone does; and (c) how the observed clinical improvement might affect the caregiver burden, as previously shown in other studies.^19^

## AUTHOR CONTRIBUTIONS

**Marianna Contrada:** Conceptualization; data curation; methodology; supervision; writing – original draft. **Federica Scarfone:** Methodology; writing – review and editing. **Antonella Iozzi:** Formal analysis. **Simone Carozzo:** Formal analysis. **Martina Vatrano:** Formal analysis. **Maria Grazia Nicoletta:** Data curation; investigation. **Giuseppe Nudo:** Investigation. **Maria Quintieri:** Investigation. **Paolo Tonin:** Investigation; resources; supervision. **Antonio Cerasa:** Methodology; supervision; writing – original draft; writing – review and editing.

## FUNDING INFORMATION

This study has not received any financial support from any organization.

## CONFLICT OF INTEREST STATEMENT

The authors have no relevant financial or non‐financial interests to disclose.

## ETHICS STATEMENT

The study was approved by the Ethical Committee of the Central Area Regione Calabria of Catanzaro (Protocol n. 346. October 21, 2021). All procedures performed in this study involving human participant were in accordance with the ethical standards of the institutional and/or national research committee and with the 1964 Helsinki Declaration and its later amendments or comparable ethical standards.

## CONSENT

Written informed consent was obtained from the patient to publish this report in accordance with the journal's patient consent policy.

## Data Availability

Datasets associated with the present study are available upon reasonable request of interested researchers.
